# The Evolving Threat of Fusarium Wilt TR4 to Small-Scale Mixed Cultivar Banana Production in the Red River Basin of Northern Vietnam

**DOI:** 10.3390/jof11090653

**Published:** 2025-09-04

**Authors:** Chung Huy Nguyen, Thi Tho Nguyen, Diane Mostert, Altus Viljoen, Elizabeth Kearsley, Guy Blomme

**Affiliations:** 1 Plant Protection Research Institute, Hanoi 11909, Vietnam; hchungvasi@yahoo.com (C.H.N.); ntho57.ppri@gmail.com (T.T.N.); 2 Department of Plant Pathology, Stellenbosch University, Stellenbosch 7602, South Africa; diane@sun.ac.za (D.M.); altus@sun.ac.za (A.V.); 3 BlueGreen Labs, 9120 Melsele, Belgium; elizabeth@bluegreenlabs.org; 4 Bioversity International, c/o ILRI, Addis Ababa P.O. Box 5689, Ethiopia

**Keywords:** Banana disease, Cavendish, disease spread, *Musa* spp., Pisang Awak, varietal resistance

## Abstract

Fusarium wilt (Foc) TR4 was first reported in Northern Vietnam in 2018. Since then, it has rapidly spread across most northern provinces along the Red River basin banana production landscapes, impacting Cavendish (*Musa* AAA genome) production. The other main banana cultivars which are widely grown in this production zone are Pisang Awak (*Musa* ABB genome) and Pisang Mas (*Musa* AA genome). Field surveys were conducted in 2022/2023 across this banana production region to assess pathogen spread from Cavendish monocropping systems into adjacent smaller-scale mixed cultivar systems. Across 130 sites, a total of 210 banana pseudostem tissue samples were collected from symptomatic Cavendish, Pisang Awak and Pisang Mas plants. Foc TR4 incursions into mixed small-to-mid-sized Cavendish–Pisang Awak plantations were confirmed, and the pathogen was also recorded in Pisang Awak plantations and backyard gardens that did not contain any Cavendish mats. A screenhouse-based Foc TR4 screening trial including seven commonly cultivated *Musa* varieties in Northern Vietnam indicated that Pisang Awak and Pisang Mas are susceptible to the pathogen. While Pisang Awak, an important local variety, is known to be susceptible to both Foc Race 1 and TR4, recent field observations suggest a limited susceptibility of Pisang Awak to Foc TR4 in mixed cultivar plantation settings. Local farmers similarly reported observing reduced susceptibility, with several having already replanted TR4-affected Cavendish fields with Pisang Awak as part of their disease management strategy. No infections were observed on field-grown Pisang Mas plants in TR4-affected mixed banana cultivar production landscapes. These results and insights provide solutions for the revival of TR4-affected Cavendish production fields or landscapes, through the cultivation of less susceptible local cultivars. In addition, the introduction, validation and scaling of Formosana (i.e., GCTCV-218, a Cavendish somaclone with moderate resistance to Foc TR4) should be envisaged.

## 1. Introduction

Fusarium wilt, caused by the soil-borne fungus *Fusarium oxysporum* f. sp. *cubense* (Foc), is a major disease affecting banana (*Musa* spp.) plants worldwide. Foc is classified into three races based on its pathogenicity to different banana cultivars, with Foc tropical race 4 (TR4) being particularly devastating to the Cavendish banana subgroup, in addition to other dessert and cooking banana varieties [1,2,3,4]. Over the past decade, Foc TR4 has been reported in numerous countries in Asia, including India, Pakistan, Indonesia, Malaysia, the Philippines, Taiwan, various Greater Mekong Sub-Region countries [5,6,7,8], the Northern Territory, and Queensland in Australia [9]. In addition, the disease emerged in the Middle East including Israel, Oman and Jordan [10,11,12], on the South-East African coast (Mozambique, Mayotte and the Comoros Islands) [13,14,15], and more recently in Latin America (Colombia, Peru and Venezuela) [16,17,18].

The decline of Cavendish production in southern China due to Foc TR4 has led to an expansion of large-scale Cavendish production in the Greater Mekong Sub-Region over the past decade [7]. Vietnam, a rising banana exporter in the region, currently exports 430 kt annually (2023), primarily to China, marking a 6% increase from the previous year [19]. As such, Vietnamese banana production has surged from 1.8 to 2.5 Mt between 2012 and 2022 [20]. Chinese private companies have facilitated this expansion by investing in banana production in Myanmar, Laos and Vietnam. This expansion was unfortunately associated with cross-border movements of planting materials and used farm equipment. The movement of Foc TR4-infected planting materials and contaminated soil from affected areas in China has inadvertently contributed to the further dissemination of the pathogen [10]. Since 2018, the fungus was reported in Laos [7,21], Vietnam [7,22], Myanmar, Cambodia [7], and Thailand [23].

Since the initial detection of Foc TR4 on Cavendish monoculture farms in the northern Vietnamese provinces of Hanoi, Hung Yen and Lao Cai in 2014 and 2015 [22], further spread has been reported. Thi et al. (2022) [24] estimated that, at the time of their survey in 2018–2019, only 10% of Fusarium wilt in Northern Vietnam was due to Foc TR4. They attributed this lower prevalence to the dominance of small-scale banana production systems in the region, contrasting with large-scale monoculture Cavendish production. However, Chittarath et al. (2022) [25] established that Foc TR4 had already moved into small-scale Cavendish farms and backyard gardens, and that the pathogen had further spread to the northern provinces of Lai Chai, Phu Tho, Hai Phong and Vinh Phuc. Pham et al. (2024) [26] subsequently confirmed the widespread establishment of Foc TR4 throughout nearly all provinces in the Red River Delta region, affecting Cavendish production.

In Northern Vietnam, the pathogen spreads across fields, landscapes and regions through garden tools, infected planting materials, waterways and rivers, flooding events and through the movement of soil particles adhered to boots, shoes, clothes, farm machinery, truck/car/motorcycle/bicycle tires, skin, hooves, and paws of various domesticated and wild animals.

Foc TR4 also poses a substantial threat to other local banana varieties, which are widely consumed in Northern Vietnam. Alarmingly, Thi et al. (2022) [24] detected Foc TR4 in the ABB Tay banana cultivar (Pisang Awak), an important local variety. However, Chittarath et al. (2022) [25] did not identify Foc TR4 infections of Pisang Awak (ABB genome group) throughout their extensive survey. Pisang Awak was affected by a diverse set of VCGs belonging to Foc race 1 (R1) (VCGs 0123, 0124/5, 01218 and 01221), which are well established in the region.

Chittarath et al. (2022) [25] however warned of ongoing and possible future spread of Foc TR4 to other *Musa* cultivars, both in mixed cultivar and mono-cropped arrangements.

Resistance evaluations of banana genotypes to Foc R1 and TR4 under glasshouse and field conditions demonstrated the susceptibility of Pisang Awak to both Foc R1 and TR4 [27]. While most surveys and risk assessments list Pisang Awak among Foc TR4-affected varieties [28,29], more recent findings indicate variation at the subgroup and/or clone level. Thangavelu et al. (2025) [30], for example, observed that among four Pisang Awak accessions, the cultivar ‘Namwa Khom’ displayed immune reactions in field trials and moderate resistance in pot experiments against Foc TR4. Preliminary results from a Fusarium wilt TR4 screening trial conducted by the Direction de l’Alimentation, de l’Agriculture et de la Forêt (DAAF)-Mayotte indicate that the ABB cultivars Pelipita and Pisang Awak/Kayinja express partial resistance to Foc TR4 (L. Larroche, comm., DAAF, Mamoudzou, Mayotte). These results confirm that Pisang Awak’s response to Foc TR4 might depend on the specific clone, and likely also the Foc TR4 strain involved. Given this variability, clarifying the reaction of the Pisang Awak accession cultivated in Northern Vietnam to the local Foc TR4 strain is both warranted and urgent.

Considering the omnipresence and ongoing expansion of banana cultivation, as large plantations, smaller fields and backyards, this study aimed to assess the potential spread of Foc TR4 from Cavendish-based mono-cropped production systems into adjacent small-to-mid-scale mixed cultivar farms along the Red River basin in Northern Vietnam, as such an expansion would have significant economic and food security implications for smallholder farmers. To achieve this, an extensive field survey was conducted in nine provinces along the Red River basin, as a follow-up of the country-wide survey by Chittarath et al. (2022) [25]. Additionally, pathogenicity experiments were performed to evaluate the susceptibility to Foc TR4 infection of six local *Musa* varieties (including Cavendish and Pisang Awak) and Formosana, a Giant Cavendish Tissue Culture Variant (GCTCV-218) known for its moderate resistance to Foc TR4.

## 2. Materials and Methods

### 2.1. Field Surveys

**Figure 1:**
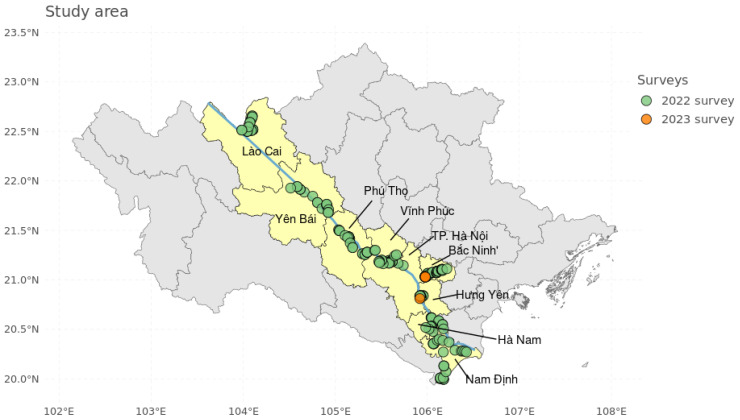
Survey area along the Red River basin in Northern Vietnam. Main survey locations assessed in November 2022–January 2023 are indicated by green points. Sites with higher sampling density visited in October 2023 are indicated in orange. The blue line represents the Red river.

A field survey was performed between November 2022 and January 2023, in banana production landscapes along the Red River basin, across 9 northern provinces of Vietnam, to assess the geographical spread and diversity of Fusarium wilt (Figure 1). The survey focused on smaller-scale predominantly mixed cultivar production systems, such as small-to-mid-sized plantations, backyard gardens and communal lands, in production zones adjacent to Foc TR4-infected Cavendish dominated monoculture plantations/production landscapes. The location of these infected Cavendish monoculture plantations was determined during a 2020/2021 field survey [25]. By driving through these partially infected banana production landscapes and talking to local farmers, the survey team identified and visited mixed/local cultivar fields with typical symptoms of Fusarium wilt. In these regions, the smaller-scale production landscapes are dominated by Cavendish [*Musa* AAA], *Musa* ABB type cultivars [e.g., ‘Pisang Awak’], and *Musa* AA cultivars [e.g., ‘Pisang Mas’]. Banana pseudostem tissue samples were collected from symptomatic plants of the various affected cultivars at each site. A total of 210 samples were collected across 130 sites, including 78 small-to-mid-sized plantations, 31 backyard gardens, 9 communal lands within villages, and 12 locations along main roads outside of villages. The number of samples was determined by the specific study objectives. Samples were collected using standard collection protocols [31]. Subsequently, morphological and molecular characterization of Foc samples were carried out at Stellenbosch University, Republic of South Africa.

Farm owners of small-to-mid-sized plantations and backyard gardens were interviewed to characterize their production systems. The questions focused on cultivars grown on the farm, age of the field, source and types of planting materials used, occurrence of water logging, type of cropping system and the first occurrence of symptoms.

In October 2023, 3 additional farms were surveyed near Hanoi (2 in Hanoi province, and 1 on the border of Hanoi and Hung Yen province) to confirm observations made during the initial survey of the apparent limited susceptibility of Pisang Awak to Foc TR4 in mixed cultivar [Cavendish and Pisang Awak] fields with the presence of both Foc R1 and TR4. At these 3 farms, both Cavendish and ABBs were grown together/adjacent to each other, and both varieties showed typical Fusarium wilt symptoms. A high number of samples were collected from symptomatic plants of each cultivar to investigate field-level Foc diversity and the Foc race associated with each cultivar. A total of 57 samples were collected from 19 Cavendish and 38 Pisang Awak plants.

### 2.2. Plant Sample Analysis

Fungal isolation: From each sample, four vascular strands of 5 mm each were cut and plated onto potato dextrose agar (PDA) supplemented with streptomycin (4 mg/L). Thereafter, cultures were incubated for 7 days at 25 °C, purified and single spored. Only samples that resembled *Fusarium* in culture were stored in glycerol at −80 °C and on carnation leaf agar (CLA) slants at 4 °C in the culture collection of the Department of Plant Pathology at Stellenbosch University, South Africa. Of the 267 samples only 178 samples had the typical spore morphology of *Fusarium*, on which molecular characterization and VCG analysis were subsequently carried out.

DNA extraction: For DNA-based studies, the isolates were grown on PDA at 25 °C for 5 days. Fungal mycelium of each isolate was harvested by scraping it off the surface of the growth media with a sterile scalpel and depositing into 2 mL Eppendorf tubes and lyophilized in a VirTis BenchTop Pro freeze dryer (SP Scientific, Warminster, PA, USA). DNA extractions were performed as described by González-Mendoza et al. (2010) [32], with minor modifications explained in the following section. Approximately 100 mg of freeze-dried mycelia were placed in a 2 mL Eppendorf tube with glass beads and shaken in a ball mill (Retsch, Haan, Germany) for 5 min at 30 Hz. This was followed by the addition of 0.5 mL extraction buffer (100 mL L^−1^ 0.5 M EDTA at pH 8, 100 mL L^−1^ 1 M Tris-HCL at pH 8, 166 mL L^−1^ 3 M NaCl, 12.5 mL L^−1^ 20% SDS), and vortexed for 15 s. A total of 0.5 mL of chloroform/phenol/isoamyl alcohol (25:24:1) was added to each sample, and the samples were incubated at 65 °C for 5 min. The samples were then cooled to room temperature and centrifuged at 11 600 revolutions per minute (rpm) for 5 min. Three hundred µL of the supernatant was transferred to 1.5 mL Eppendorf tubes, followed by the addition of 300 µL refrigerated (−20 °C) 100% ethanol. The samples were mixed by pipetting and incubated at −20 °C for 30 min to allow the DNA to precipitate. The samples were then centrifuged at 11,600 rpm for 10 min, and the supernatant was discarded. The pellet was washed twice by centrifugation with 0.5 mL, refrigerated 75% ethanol for 5 min at 11,600 rpm, and the supernatant discarded. The pellets were left at room temperature to air-dry and were resuspended in 60 µL TE buffer (10 mL/L 1 M Tris at pH 8.0 and 2 mL/L 0.5 M EDTA). Samples were incubated with 3 µL RNase (10 mg/mL) at 65 °C for 10 min to remove RNA. The quality and quantity of DNA was determined with a NanoDrop Nd-1000 Spectrophotometer (Thermo Fisher Scientific, Wilmington, DE USA) and stored at −20 °C until use.

Molecular identification with PCR: Molecular identification for Foc was performed as reported in Chittarath et al. (2022) [25] with a few modifications which are explained in the next paragraph.

DNA was first tested with a Foc TR4-specific PCR assay as described by Dita et al. (2010) [33]. If negative for Foc TR4 PCR, it was tested with Foc Clade A specific PCR (unpublished) to determine if samples were within any of the known VCGs in Clade A [34]. If it was negative, PCRs specific for Foc Lineage VI as described by Ndayihanzamaso et al. (2020) [35] were conducted. Isolates that were negative for all previously mentioned PCRs were subjected PCR assays specific to Foc Lineage VII, VCG 01218 and VCG 01221, respectively (unpublished).

Vegetative compatibility group (VCG) testing: To confirm the identity of all Foc isolates, VCG testing was conducted [36]. The isolates were first grown on PDA for 7 days at 25 °C. Thereafter, five replicates of the actively growing isolates were sub-cultured onto minimal media (MM) supplemented with 1–3% KClO_3_ and incubated for 7–21 days at 25 °C to generate ClO_3_-resistant mutants. The ClO_3_-resistant mutants were then transferred to MM slants and were incubated for an additional 7–10 days. Sparse mycelial growth colonies were considered nitrate non-utilizing (nit)-mutants and typed as nit-1, nit-3 or Nit-M mutants [36]. The VCG identity of Foc isolates from Vietnam were then determined by pairing nit-1 and nit-3 mutants with Nit-M testers of known VCGs or vice versa. The samples that tested positive for Foc TR4 were tested against VCGs 01213, 01216, and 01213/16. All the isolates that tested positive for the Lineage VI-specific PCR were tested against VCGs 0124, 0125, 0128, 01212, 01220, and 01222. VCG testing was also performed to confirm PCRs specific to VCG 01218. Heterokaryons were identified when anastomosis of complementary hyphae resulted in a fluffy wild-type growth [36]. When the nit-1 and nit-3 mutants of a particular isolate did not pair with its own Nit-M mutants, it was considered as heterokaryon self-incompatible.

### 2.3. Screenhouse-Based Screening Trial

Preparation of plant material: Seven *Musa* varieties were screened to assess Foc TR4 susceptibility, including the accessions Green Cavendish (Tiêu xanh, AAA), Pink Cavendish (Tiêu Hồng, AAA), Pisang Awak (Tây Thái) (ABB), Red banana (Chuối đỏ) (AAA), Pisang Mas (Chuối ngự) (AA) and ‘Chuoi sap’ (ABB), all being popular and widely grown in Vietnamese production systems. In addition, Formosana, a Giant Cavendish Tissue Culture Variant (GCTCV-218) developed for its resistance to Foc TR4 [37] was evaluated. Plantlets of the common accessions were sourced from a tissue culture plantlet distribution center based in Hanoi, while the Formosana plantlets were sourced from the Taiwan Banana Research Institute (TBRI). All planting material were sourced as rooted tissue culture-derived plantlets, which were hardened in trays before the trial. No other *Musa* accessions were available from tissue culture distribution centers in Hanoi.

Plantlets with a height of 15 cm were potted, on 8 December 2023, into plastic pots with a truncated cone shape and diameter of 15 cm (circular top) and 20 cm (circular bottom), and a height of 18 cm. These pots contained soil comprising 50% forest soil and 50% Peat Moss BVB Substrates (Netherlands, contains 50% black peat and 50% white peat, pH 5–6). All plantlets were hardened to the age of four months in a Plant Protection Research Institute (PPRI) screenhouse in Hanoi.

Preparation of inoculum and infection: Inner pseudostem samples were collected in an infected Cavendish field from an Foc TR4-infected Cavendish plant with multiple yellow leaves and ample discolored vascular strands in its pseudostem. The fungal pathogen was isolated on Potato Dextrose Agar (“PDA”) medium and subsequently cultured on steam-sterilized millet seeds for mass multiplication of Foc conidia as described by Ndayihanzamaso et al. (2022) [38].

For each of the seven accessions, 15 plants in three reps of five plants were inoculated on 27 February 2024, i.e., 12 weeks after potting, while 5 plants of each accession served as the un-inoculated control, as described in Dita et al. (2021) [39]. The Green and Pink Cavendish cultivars served as the highly susceptible control plants. At the time of inoculation, plants were lifted out of their planting bags leaving about 30% of the potting soil in the bag [38]. The millet seed inoculum (30 g per plant) was then placed on the remaining soil, and the plants were replanted onto the bottom soil and inoculum. This method allows for the banana roots to be in direct contact with the millet seed inoculum. Plantlets were regularly watered as needed.

Monitoring and scoring disease development: The appearance of leaf yellowing and wilting symptoms was monitored on a weekly basis. When at least 80% of the Green and Pink Cavendish plants (which were considered as having the highest level of susceptibility to Foc TR4) had clear leaf yellowing symptoms on outer leaves (i.e., at 16 weeks after inoculation), all trial plants were assessed for internal rhizome/corm tissue discoloration and leaf symptoms.

Rhizomes (i.e., corms) of each plant were sliced open transversely, starting from the rhizome basal section (i.e., root side), with additional transversal cuts made towards the rhizome’s apical section. A total of 4 transversal cuts were made on each rhizome. As tissue discoloration often varies across a corm, the corm transversal section with maximum tissue discoloration was selected for scoring. This section is often located about a third upwards into the rhizome starting from the basal corm region.

Leaf and rhizome symptom development were scored by 2 independent researchers. Leaf symptoms were scored using the following 1–5 level scale: 1 = no leaf yellowing symptoms, 2 = initial yellowing mainly on the oldest/outer leaves, 3 = yellowing of all the outer/oldest leaves with some discoloration of younger/inner leaves, 4 = all leaves had intense yellowing, and 5 = the plant died [39]. Rhizome tissue discoloration was scored according to a 1–6 level scale: 1 = no symptoms on the transversally cut rhizome surface, 2 = only small, discolored spots, 3 = <1/3 of rhizome discolored, 4 = 1/3 to 2/3 of rhizome discolored, 5 = >2/3 of rhizome discolored, 6 = entire rhizome discolored [38].

## 3. Data Analysis

The presence of Foc Races in various provinces, stratified by banana production systems, was determined using descriptive statistics. Farmers’ interview responses, characterizing their banana production system and disease occurrence, were summarized as the percentage of farmers giving the same response. Disease symptom progression scores for cultivars in the screening trial were assessed through the Kruskal–Wallis test combined with a pairwise comparison (α = 0.05) using the Mann–Whitney U (Wilcoxon rank sum) test (following rejections of homoscedasticity to perform an analysis of variance). The effect size for Kruskal–Wallis test is reported as the eta squared based on the H-statistic. Data analysis was performed using the R Statistical software version 4.4.0 [40].

## 4. Results

### 4.1. Field Survey

Of the 130 mixed/local banana cultivar sites with symptomatic plants sampled, 96 sites were confirmed as being infected by Fusarium wilt, including 63 small-to-mid-sized plantations, 23 backyard gardens, 4 communal lands in villages, and 6 plants alongside roads.

**Figure 2:**
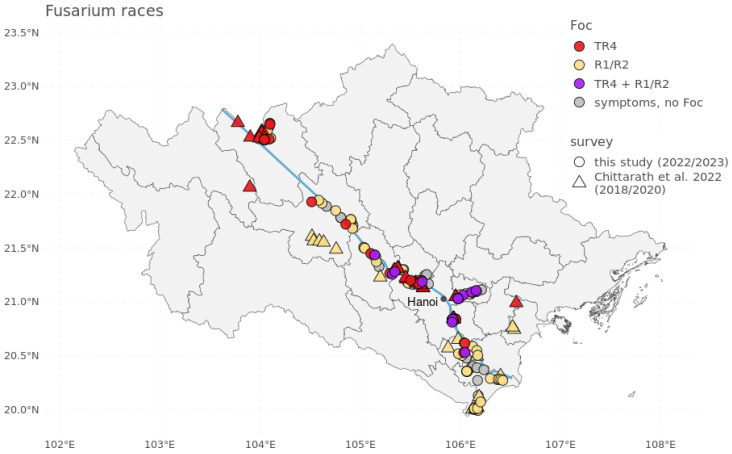
Observations of *Fusarium oxysporum* f. sp. *cubense* (Foc) TR4 and Foc R1/R2. Fields surveyed in this study are indicated as circles, while fields surveyed in the Chittarath et al. (2022) [25] survey are marked as triangles. gray points show fields with banana plants showing the traditional symptoms of wilt, but which were not identified as Fusarium wilt; red points denote sites where Foc TR4 was positively identified; yellow points indicate sites where Foc R1/R2 was positively identified; and purple points show sites where both Foc TR4 and R1/R2 were positively identified. The blue line represents the Red river.

Overall, 53 sites were uniquely affected by Foc R1/R2, 34 sites by Foc TR4, and 9 sites by both Foc TR4 and R1/R2 (Figure 2). Foc TR4 specifically has spread to small-scale mixed banana cultivar production systems (4 backyard gardens and 39 small-to-mid-sized plantations) in the provinces Lao Cai, Yen Bai, Phu Tho, Vinh Phuc, Ha Noi, Bac Ninh, Hung Yen and Ha Nam (i.e., all surveyed provinces except for Nam Dinh).

The surveyed banana production landscape along the Red River was dominated by Cavendish and Pisang Awak. Cavendish was only affected by Foc TR4, recorded in 30 small-to-mid-sized plantations and one backyard garden (Table 1). Pisang Awak was mainly affected by Foc R1/R2, although a substantial number of infections by Foc TR4 were also recorded (Table 1). Pisang Awak infected with Foc TR4 was recorded in 13 sites (3 backyard gardens and 10 small-to-mid-sized plantations), spread across 7 provinces (Lao Cai, Yen Bai, Phu Tho, Vinh Phuc, Bac Ninh, Hung Yen and Ha Nam). Pisang Awak remains mainly affected by R1/R2, recorded on 67 sites including the communal lands in villages and along roads (Table 1). In 64 of these sites, Pisang Awak was affected by Foc R1/R2 VCGs 0124/5/8/12/20/22 (Table 2). In one farm in Phu Tho, a second host cultivar Pisang Mas was also infected with R1/R2 VCG 0124/22. Two farms (in Ha Noi and Hung Yen, respectively) had Pisang Awak plants infected with Foc strains belonging to the Foc Lineage VI, but these were not compatible to known VCGs in this Lineage. At these farms the presence of Foc R1/R2 VCGs 0124/5/22 was also confirmed. In the remaining farms, one site in Nam Dinh had Pisang Awak plants infected by VCG 01218, while in two sites in Lao Cai and Yen Bai, respectively, Foc Lineage VI isolates not compatible to known VCGs in this lineage, affecting Pisang Awak, were recorded.

At nine sites, both Foc TR4 and R1/R2 infected banana plants were recorded within the same field (Figure 2, Table 1). In five plantations and in one backyard garden, Pisang Awak plants were affected by Foc R1/R2, while Cavendish plants were affected by Foc TR4. At three plantations, Foc TR4 and Foc R1/R2 had both affected Pisang Awak plants.

At the three banana plantations surveyed in 2023 in which a larger number of samples were collected per farm, the apparent limited susceptibility of Pisang Awak to Foc TR4 in mixed cultivar [Cavendish and Pisang Awak] fields with the presence of both Race 1 and TR4 was confirmed. Across these farms, only a single sampled plant of Pisang Awak was infected by Foc TR4, while 21 Pisang Awak plants infected by Foc R1/R2 were recorded. Cavendish was uniquely affected by Foc TR4 (18 identified plants).

As reported by the farmers of backyard gardens and small-to-mid-sized plantations with TR4-confirmed infections, symptoms were first observed on average 1.1 ± 0.4 years ago (with respect to the survey date in November 2022–January 2023). This ranges from 1.5 ± 1.0 years ago in Lao Cai to 0.5 ± 0 years ago in Yen Bai, although no significant difference is found between reported incursion dates across Provinces.

Across the sampled locations, Foc TR4 and R1/R2 are confirmed in backyard gardens and small-to-mid-sized plantations, where farmers either use suckers or tissue culture plantlets as planting material (71% and 10% of affected farms), where banana is cultivated as monocrop or intercrop (79% and 15% of affected farms), and in both waterlogged and well-drained fields (9% and 84% of affected farms). While the sampling design was not set up to differentiate the impact of farm management and type on the presence of Fusarium wilt, the field survey indicates Foc TR4 and R1/R2 have spread to various farm types (Table 1).

### 4.2. Screening Trial

All seven banana accessions developed internal rhizome symptoms by week 16, when all plants were assessed for both internal and leaf symptoms, following initial soil inoculation with Foc TR4. Only one Formosana plant showed no internal rhizome symptoms. Overall, Formosana had the lowest rhizome discoloration score (*p* < 0.001), while all other accessions exhibited high and indistinguishable discoloration scores (Table 3).

**Figure 3:**
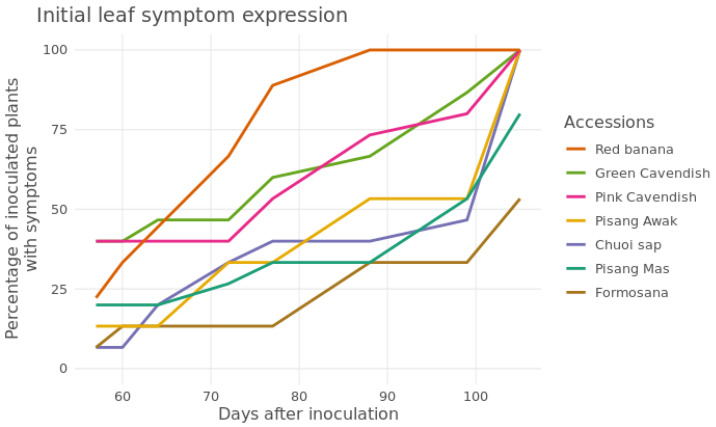
Number of inoculated plants on which initial leaf symptoms could be discerned, and symptom progression over a 105 day period, during the Foc TR4 screenhouse-based screening trial. The local Vietnamese names for each accession are listed in Table 3.

As external leaf symptoms lag symptom development in the rhizome, leaf discoloration scores were generally lower. Leaf discoloration of the accessions Formosana, Pisang Mas, Pisang Awak and Chuoi sap were still in the early symptom development stages (i.e., leaf scores of 1.8–2.1) by week 16, while the Cavendish accessions and Red banana had more severe leaf symptoms (i.e., leaf scores of 2.5–3.1) (Table 3). The number of plants which presented initial symptoms of Fusarium wilt during the trial period was consistently higher for the latter three accessions (Figure 3).

In line with field survey observations, the screenhouse data shows a similar pattern regarding aboveground symptoms, with Pisang Awak and Pisang Mas clearly showing delayed and less severe leaf symptoms compared to the highly susceptible Cavendish accessions.

## 5. Discussion

The extensive field survey along the Red River basin banana production landscapes indicates continued spread of Foc TR4 within the northern provinces of Vietnam from where it was first reported [22]. The survey identified first incursions of Foc TR4 into the Bac Ninh and Hung Yen provinces, west of Ha Noi, and identifies an increasing number of affected farms in affected northern provinces compared to our previous surveillance conducted between 2020 and 2021 [25], only two years prior. Foc TR4 is hereby well established in these banana production landscapes, and the pathogen is continuing to spread.

By sampling symptomatic plants in all identified affected fields, the survey identified two areas with a high occurrence of Foc TR4. Firstly, Foc TR4 is well established in the northern province of Lao Cai, in banana production regions bordering southern China. Secondly, several clusters of Foc TR4-affected production regions were identified in the Red River delta, with a high occurrence along the bordering regions of Ha Noi, Vinh Phuc and Phu Tho, in the province Bac Ninh, and along the borders of Ha Noi and Hung Yen. Further geographically scattered detections of Foc TR4 were made throughout the delta. While the initial incursion of Foc TR4 might have been related to the expansion of Cavendish mono-cropped cultivation in response to Chinese demand [7,10], the dispersal of Foc TR4 in Northern Vietnam at present likely continues through local and regional factors. Local trade of Cavendish suckers likely initiated the spread of Foc TR4 into the small-to-mid-sized Cavendish plantations where most infections have been identified. This observation is in line with Chittarath et al. (2022) [25]. Worryingly, in this study, Foc TR4 infections were also identified in mixed cultivar small-to-mid-sized Cavendish—Pisang Awak plantations, and in Pisang Awak plantations and backyard garden without Cavendish cultivation. As such, two widely grown banana varieties are currently affected, and continued trade of planting material will continue the spread of Foc TR4 to smallholders and households cultivating bananas in backyard gardens. The rapid dissemination of Foc TR4 in this region is likely exacerbated through water movement from the Red River and its many tributaries running through infected (Cavendish) production zones. Halting the spread of Foc TR4 is practically not possible, but focused action to minimize inoculum levels and continued cultivation via partially resistant and resistant cultivars could safeguard production. Critically, substantial infections of Pisang Awak plants with Foc R1/R2 have also been recorded across the entire Red River delta (also identified in 25 and 24). Management practices and cultivar selections therefore need to take the presence of both Foc strains into account.

Pisang Awak is a known susceptible variety to both Foc R1 and TR4 [27,41], although it has shown partial resistance to Foc TR4, for example, under field conditions in the Philippines ([42] under local name Kluai Namwa). Indeed, while Foc TR4 infections of Pisang Awak were recorded in Northern Vietnam, farmers reported an apparent limited susceptibility of Pisang Awak to TR4 in mixed cultivar [Cavendish and Pisang Awak] fields, contradicting common assumptions that varieties that are highly susceptible to R1/R2 are also susceptible to Foc TR4. Several farmers further reported that they had already replaced their Foc TR4 affected Cavendish fields with Pisang Awak, as they had observed far less infections in this cultivar. The field surveys in mixed Cavendish–Pisang Awak plantations confirmed the limited susceptibility of Pisang Awak to Foc TR4. In addition to eradicating affected Cavendish plants, a one or two year rotation with other crops (e.g., vegetables, papaya, young trees) before replanting with Pisang Awak, and the integration of leguminous cover crops should further reduce or keep soil inoculum levels low and reduce both abiotic and biotic stress conditions.

While Cavendish and Pisang Awak dominate the *Musa* production landscapes along the Red River basin, a third locally important diploid variety, Pisang Mas, did not show Foc TR4 infections in field grown plants. Resistance (or tolerance) of Pisang Mas to Foc TR4 under both glasshouse and field conditions, and to Foc R1 under field conditions, was identified by Subbaraya et al. (2024) [27].

The seven *Musa* varieties included in the screenhouse-based screening trial all developed internal rhizome symptoms when infected with Foc TR4, with Formosana having a significantly lower rhizome discoloration score than the other six varieties. This finding is concerning, as it indicates that all varieties currently being grown by banana producers in Vietnam are susceptible to Foc TR4. Formosana, a somaclonal variant of Giant Cavendish (Giant Cavendish Tissue Culture Variant; GCTCV-218), is considered partially resistant to Foc TR4 [37], meaning it can tolerate the disease when soil inoculum pressure is low, and the plant is not under any abiotic stress condition. The low rhizome score, in addition to a relatively low leaf score of Formosana (Table 2) seems in line with its reported moderate field-level Foc TR4 resistance.

In addition, external leaf symptoms of Formosana in the small-plant screening trial were less severe and developed slower than that of the highly susceptible Cavendish cultivars. Pisang Mas, Pisang Awak and Choi Sap, also, showed reduced leaf symptoms and slow leaf symptom development. This result shows a similar trend with the contrasting field-level observations between Cavendish on one hand, and Pisang Awak and Pisang Mas on the other hand. Respectively, limited or no leaf symptoms were reported for the latter two cultivars during the field survey. Additional pot-based pathogenicity testing, including a range of inoculum levels, and longer field-level screening trials, would further help elucidate susceptibility levels.

In the absence of fully resistant economically viable banana varieties, mitigation of the impact of Foc TR4 in Northern Vietnam could rely on the cultivation of Pisang Awak and Pisang Mas in mixed-cultivar production systems, potentially including other local varieties. Similarly, the introduction of Formosana and other partially resistant varieties should be considered. Cultivating a range of partially tolerant/resistant varieties and applying disease management approaches that improve environmental conditions are critical. This includes the establishment of farms in locations with appropriate edaphic and climatic conditions, implementing proper fertilization and irrigation, and especially keeping inoculum levels low through the timely identification of early symptoms and subsequent destruction of affected plants [3].

The survey further confirmed that Foc R1/R2 is well established in Northern Vietnam [24,25]. Most of the identified Pisang Awak infections, and a single Pisang Mas infection, belonged to VCGs in Foc Lineage VI (0124/5/8/12/20/22). This confirms previous findings by Chittarath et al. (2022) [25], Mostert et al. (2017) [43] and Somrith et al. (2011) [44] on Foc R1/R2 diversity currently present in this region. The simultaneous presence of Foc R1/R2 and TR4 complicates the *Musa* variety selection to mitigate the impact of Fusarium wilt, although overall disease management practices to keep soil/field inoculum levels low remain the same.

## 6. Conclusions

Fusarium wilt TR4 has now spread from Cavendish monocultures into smallholder mixed-cultivar systems in Northern Vietnam, threatening both local and export-oriented production. While Pisang Awak and Pisang Mas are susceptible under controlled pot trial conditions, field observations suggest Pisang Awak shows limited susceptibility to Foc TR4, and Pisang Mas remained uninfected in surveyed fields. Alongside the moderate resistance of Formosana, these findings indicate that deploying less susceptible local cultivars and partially resistant somaclonal variants within diversified production systems offers a practical pathway to sustain banana cultivation and safeguard smallholder livelihoods under increasing Foc TR4 pressure.

## 7. Future Perspectives and Challenges

The rapid spread of Fusarium wilt TR4 in Northern Vietnam underscores the urgent need for integrated disease management strategies [6,45,46] that combine surveillance, resistant or tolerant cultivar deployment, and improved agricultural practices. While this study highlights the potential of local varieties such as Pisang Awak and Pisang Mas, as well as the moderate resistance of Formosana, the partial susceptibility observed in screenhouse trials suggests that no single cultivar currently offers full resistance.

Future research should focus on large-scale, multi-location and multi-season field trials, accounting for a range of environmental conditions, of these promising varieties, alongside the exploration of novel genetic resources and breeding strategies for durable resistance. Future research streams should also include risk of spread mapping to pin-point locations for integrated disease management and continued surveillance interventions, and socio-economic analyses to gauge adoption feasibility of recommended cultivars. These additional studies would help translate the promising varietal insights into scalable, region-specific integrated management strategies.

Additionally, the concurrent presence of Foc R1/R2 and TR4 poses a complex challenge for cultivar selection and disease mitigation, warranting a better understanding of host–pathogen interactions and mixed-infection dynamics.

## Figures and Tables

**Table 1:** Foc infections identified in the various production systems, and the host plant infected per site, identified during the survey from November 2022 to January 2023. Each number indicates a separate banana field, in which one or multiple affected plants with specific VCG-host associations were identified.

Production System	Identified Infections	Cavendish		Pisang Awak		Pisang Mas
		TR4	R1/R2	TR4	R1/R2	R1/R2
Backyard garden	TR4		-	3	-	-
Backyard garden	R1/R2	-		-	19	
Backyard garden	TR4 + R1/R2	1			1	
Plantation	TR4	25	-	7	-	-
Plantation	R1/R2	-		-	24	1
Plantation	TR4 + R1/R2	5	-	3	8	
Village	R1/R2	-		-	4	
Along road	R1/R2	-		-	6	

**Table 2:** Foc diversity identified in the affected fields. Each number indicates a separate banana field, in which one or multiple affected plants with specific VCG-host associations were identified.

Fusarium Strain	VCG	Cavendish	Pisang Awak	Pisang Mas
Foc TR4	01213/16	35	14	
Foc R1/R2	0124		1	
	0124/5		19	
	0124/22	1	36	1
	0124/5/22		3	
	0124/8		1	
	0124/8/22		3	
	0124/5/8/22		1	
	0128/22		7	
	01218		1	
	Unknown		4	

**Table 3:** Average leaf and rhizome symptom scores by accessions in the TR4 screening trials carried out at the Plant Protection Research Institute (PPRI) in Hanoi. Means followed by the same letter are not significantly different (Kruskal–Wallis and Wilcoxon rank sum; *p* > 0.05). Standard deviations are provided.

Accession	Local Name	Leaf Score	Rhizome Score
Red banana	Chuối đỏ	3.1 ± 0.7 b	5.2 ± 0.8 b
Green Cavendish	Tiêu xanh	2.5 ± 0.9 ab	5.6 ± 0.7 ab
Pink Cavendish	Tiêu Hồng	2.5 ± 0.9 ab	5.6 ± 0.5 ab
Pisang Awak	Tây Thái	2.1 ± 0.3 a	5.2 ± 0.8 b
Chuoi sap		1.9 ± 0.3 a	5.8 ± 0.6 a
Pisang Mas	Chuối ngự	1.9 ± 0.3 a	5.6 ± 0.4 b
Formosana		1.8 ± 0.4 a	3.2 ± 1.4 c
*p*-value		<0.001	<0.001
eta-squared[H]		0.230	0.316

## Data Availability

The raw data supporting the conclusions of this article will be made available by the authors on request.
